# Zika virus during controlled ovarian hyperstimulation

**DOI:** 10.5935/1518-0557.20190022

**Published:** 2019

**Authors:** Beuy Joob, Viroj Wiwanitkit

**Affiliations:** 1 Sanitation 1 Medical Academic Center, Bangkok Thailand; 2 Honorary professor, dr DY Patil University, Pune, India

Dear Editor, we read the publication on “Case report of Zika virus during controlled ovarian hyperstimulation: results from follicular fluid, cumulus cells and oocytes” with a great interest. Filho *et al*. (2019) noted that “*This is the first report in the literature analysing ZIKV in the follicular fluid, cumulus cells, and oocytes*.” Indeed, the identification of the virus in follicular fluid has been mentioned for a long time (Washington *et al*., 2016; Taitson *et al*., 2017). In animal model, the existence of the virus in oocytes is already reported (Broughton *et al*., 2017). Therefore, the report by Filho *et al*. (2019) should not be the first report.
